# The bovine tuberculosis cluster in north County Sligo during 2014–16

**DOI:** 10.1186/s13620-018-0135-z

**Published:** 2018-11-28

**Authors:** Rob Doyle, Tracy A. Clegg, Guy McGrath, Jamie Tratalos, Damien Barrett, Ada Lee, Simon J. More

**Affiliations:** 1 Department of Agriculture, Food and the Marine, Backweston Administration Building, Stacumny Lane, Celbridge, Co. Kildare W23 X3PH Ireland; 2 Centre for Veterinary Epidemiology and Risk Analysis, UCD School of Veterinary Medicine, University College Dublin, Belfield, Dublin, D04 W6F6 Ireland; 3 Cyberport, Pokfulam, Hong Kong Island, Hong Kong

**Keywords:** Bovine tuberculosis, Ireland, Control, Eradication, Outbreak, Coordination

## Abstract

**Background:**

Bovine tuberculosis (bTB, caused by infection with *Mycobacterium bovis*) is endemic in the Irish cattle population, and the subject of a national eradication programme since the late 1950s. During 2014, a substantial area-level bTB outbreak developing in north County Sligo, necessitating the need for an enhanced response. This paper describes this outbreak, the response that was undertaken and some lessons learned.

**Results:**

In the north Sligo area between 2014 and 2016, 23 (31.9%) of restricted herds had 4 or more reactors to the single intradermal comparative tuberculin test (SICTT)/animals with bTB lesions disclosed during the restriction, and the majority (55.5%) of test-positive animals were identified as standard reactors to the SICTT. The herds restricted during 2014–16 were typically larger than other herds in the study area and introduced more animals during 2013. *M. bovis* was also detected in local badgers, but not deer.

**Conclusion:**

This paper describes a substantial outbreak in north County Sligo over a 3-year period. A coordinated area-based approach was a key feature of the outbreak, and substantial resources were applied to bring the outbreak under control. No definitive source was identified, nor reasons why a substantial number of herds were infected over a relatively short period. A coordinated regional approach was taken, and a number of lessons were learned including the need for urgency, for a team-based approach, for a consistent message when dealing with the public, for an area-based approach, for a degree of flexibility for the breakdown manager, and for molecular tools to assist in answering key questions relating to the source and spread of *M. bovis* to many herds during this bTB outbreak.

## Introduction

Bovine tuberculosis (bTB, caused by infection with *Mycobacterium bovis*) is endemic in the Irish cattle population, and the subject of a national eradication programme since the late 1950s [1]. The eradication programme targets both cattle and wildlife, taking into account the proven role of wildlife in the epidemiology of *M. bovis* in the Irish cattle populations [2]. Trends in time and space indicate ongoing improvement, both with respect to herd incidence [3, 4] and herd recurrence [5, 6]. Nonetheless, there are multiple ongoing challenges, including the emergence of areas of local persistence (‘hot-spot’ areas). These hot-spot areas, which may persist over many years, are a key temporo-spatial feature of bTB in cattle in Ireland [7].

Between January and June 2014, six herds in north County Sligo were restricted following identification of bTB infection, including four herds that were categorised as low risk (i.e. with a single reactor animal in each), and two as higher risk (≥ two reactors), involving three and four reactors respectively. In July 2014, two further herd restrictions were imposed, including one herd with 36 reactors at a reactor retest. Contiguous testing of these herds was prioritised. During August 2014, nine further restrictions were imposed, a high rate of bTB lesions was identified in single intradermal comparative tuberculin test (SICTT) reactor animals and parallel interferon (IFN)-γ testing was introduced. At this point, it was clear that a very significant area-level bTB outbreak was developing in north County Sligo, necessitating the need for an enhanced response.

This paper describes this area-level bTB outbreak, which occurred in north County Sligo during 2014–16, the response that was undertaken and some lessons learned.

## Materials and methods

### Study period, area and herds

The study period was from 1 January 2014 until 31 December 2016.

The north County Sligo study area consists of seven District Electoral Divisions (DEDs), U101-U109 but excluding U103 and U108 (DEDs are low-level legal administrative divisions in Ireland used by the Department of Agriculture, Food and the Marine). It is a distinct geographical area bordered to the west by the Atlantic Ocean and the east by the Dartry mountains and runs north to the Co. Leitrim border and south almost to Sligo town. The quality of agricultural land is variable. There are some large dairy herds in good limestone country in the south of the area close to the coast, as well as extensive areas of peat bog close to the slopes of the Dartry mountains. Most of the cattle population are in small suckler herds. Some herd owners also graze sheep, although this is not a major enterprise in this area. During the study period, but not before, the study area was extended to include one further herd in DED L121 that was considered by the Sligo District Veterinary Office (DVO) to be part of the ‘high-risk area’. This herd had its home fragment in County Leitrim, but a high-risk bTB restriction on rented land in the study area.

Two definitions of study area were used. The first definition was that used by field staff to allocate herds to the relevant DEDs. Herd numbers are allocated to a herd based on the location of the home farm. A second definition of study area was based on the location of land parcels, given that Irish farms are very fragmented and may have parcels of land within several DEDs. Here, we identified all herds with cattle present at the end of 2013, 2014, 2015 or 2016 plus any additional herds with registered inward cattle movements or births during 2014–16.

### Cattle

- *The data*

The data sources used in this study include:

- the Animal Health Computer System (AHCS), which holds records of all tuberculin testing of herds and animals since 1989 and laboratory testing results from the national abattoir surveillance programme,
- the Animal Identification and Movement system (AIM), with records of calf registrations since 1998 and cattle movements (farm-to-farm, via a market, exports, imports and to slaughter) and on-farm deaths in Ireland since 2000,
- the Land Parcel Information System (LPIS), which is a spatial database which identifies the boundaries of farms. This database was queried in a Geographical Information System (GIS) to identify farms with any land in the study area,
- Herdfinder, which provides local office access to LPIS and is used to identify contiguous herds to TB breakdowns,
- bTB Wildlife Unit software, which is used to manage badger surveying and capturing nationally,
- the Laboratory Information Management System (LIMS), with bTB culture data for badgers and deer submitted for post mortem examination, and
- a database of all IFN-γ test results held by the Tuberculosis and Immunology Research Laboratory at University College Dublin.

b. *Restricted herds*

Herd restrictions are imposed following initial detection of bTB during field or abattoir surveillance, and these herds are generally unable to trade, except through the disposal of cattle directly to slaughter. A breakdown refers to the initial detection of bTB. When calculating herd bTB incidence, the denominator included all herds with at least one bTB test during the year and not restricted at the start of the year, and the numerator included those denominator herds with a bTB restriction starting in the year in question. There were several differences when counting herd restrictions during 2014–16 compared with prior to 2014. During 2014–16, restrictions triggered by an abattoir (factory) lesion were deemed to start once an animal was identified as infected at slaughter. With historical restrictions (that is, prior to 2014; when data were not available on the date that a lesioned animal was identified at slaughter), the restriction start date was taken as the date that the whole-herd factory lesion test (test type (TT) 9A and 10A) was carried out. bTB restrictions were grouped based on the number of positive animals, whether 1, 2–3 and ≥ 4. The initial breakdown test was classified as *‘low-risk’* if conducted on unrestricted ‘low’ risk herds. All unrestricted ‘low’ risk herds are tested annually with the SICTT conducted on all animals > 6 weeks of age (the annual test; test type [TT] 1). All other breakdown tests using SICTTs were defined as *‘high risk’* tests, including inconclusive retests (TT3), contiguous (TT8), high risk (TT5A/F), post de-restriction (TT7B) and factory lesion tests (TT9A and TT10A; that is, following detection of a lesion during abattoir surveillance). All herds that had a test type (TT) 9A/10A were assumed to have identified a single animal with a lesion at slaughter, this being added to the total number of reactors during the restriction.

c. *Test-positive animals*

During 2014–16, an animal was considered as being positive if they had a severe/standard positive or inconclusive reaction to the SICTT, positive to an IFN-γ test or had a bTB lesion detected at slaughter. The date of the test was taken as the first date of a positive test if they had more than one test.

At each SICTT, an animal was defined as either a standard SICTT reactor, a standard inconclusive SICTT reactor, a severe inconclusive SICTT reactor or as negative using criteria as previously described [8].

Data are presented for ‘diagnostic’ IFN-γ tests on SICTT negative animals. Each IFN-γ result was paired with an associated SICTT no more than 60 days prior to the IFN-γ test in order to check that the test was carried out on a SICTT-negative animal. Some animals that were negative to SICCT and/or the IFN-γ test were removed to slaughter as they either had clinical signs of bTB or were deemed reactor on the basis of potential future risk based on exposure to a high-risk cohort positive to SICCT and/or the IFN-γ.

### Wildlife

#### Badgers

Prior to August 2014, badgers were removed at a low intensity in the study area under licence by DAFM as part of a national bTB control strategy. Subsequently, many of the restricted herds qualified for badger removal in their vicinity [9]. Badgers culled from the environs of these herds were sent to the Sligo Regional Veterinary Laboratory (RVL) for gross post-mortem and bacterial culture. Road casualty badgers from the area were also submitted.

#### Deer

Deer populations fall under the remit of the National Parks and Wildlife Service (NPWS), and DAFM have no statutory responsibility for their management. Deer shooting with firearms is permitted under licence; the open season is generally from 01 September to 31 December each year for males and 01 November to the last day of February in the following year for females. Shooting out of season is permitted under section 42 of the Wildlife Act, 1976 when it can be proven that deer are causing damage to livestock. Following consultation with NPWS, who helped to establish contact with local hunters, a programme was put in place to conduct post-mortems on as many deer as possible from the study area. When deer were shot by hunters, they had the option of either submitting the head, heart and lungs to Sligo RVL or contacting a member of DAFM’s wildlife team, who would arrange to collect the material and deliver it to the RVL. This programme was facilitated by Sligo RVL, which carried out a detailed post mortem examination of the head, heart and lungs of the deer. Lymph nodes tissue from both the badgers and deer were cultured for *M. bovis*.

### Data analyses

Data analyses were conducted using Microsoft Excel (Microsoft Corporation, Redmond, WA, USA), SAS 9.4 (SAS Institute Inc., 2008) and STATA 14 (StataCorp LP, College Station, TX, USA).

- *Herd-level analyses*

The numbers of new bTB restrictions each month in the study area between 2014 and 2016 are presented by test type (high/low risk) and severity of the restriction. The annual incidence rate for bTB restrictions in the North Sligo area were estimated from 1989 to 2016.

The previous history of bTB restrictions between 2009 and 2013 (1–5 years prior), 2004–08 (6–10 years prior) and since 1989 (the start of available records) until 2003 were estimated for herds which were restricted between 2014 and 16 and for the remainder of herds which were tested for bTB as least annually during this 3-year period.

b. *Animal-level analyses*

The monthly number of bTB test-positive animals, between 2014 and 2016, was calculated by the type of diagnostic test that identified them as positive. For animals that were positive to a ‘diagnostic’ IFN-γ test and negative to the SICTT (conducted no greater than 14 days prior to the IFN-γ test), the bTB status at post-mortem is presented. In addition, the number of animals positive to the IFN-γ test and the number of SICTT reactors within a herd were calculated.

c. *Spatial analyses*

The location of the restricted herds during 2014–16 was mapped, after all farms had been ‘jittered’ to maintain farmer anonymity. This was done by dispersing farms randomly on a land location (areas of sea were excluded) somewhere within a 1 km radius of the centroid of the largest fragment of land for each farm. Further, a kernel density analysis [10] was conducted of all herds in Ireland with 2 or more standard reactors over the period 2014 to 2016. The grid size was set to 100 m and the kernel bandwidth to 5 km. A cut-off of 0.2 positive herds per square kilometre was selected to display only areas with significant bTB levels.

d. *Herd characteristics and movements*

Cattle movements and herd sizes were analysed using the data from the AIM database. We used data for 2013–16 comprising animal-level information on cattle movements and birth registrations. We calculated the number of inward movements made in 2013 to herds involved in the overall outbreak and compared these to the number of movements made to other herds which hold land within the North Sligo study area. 2013, the year before the start of the outbreak, was used because movement restrictions in later years would have reduced the number of movements to herds testing positive. We also used the AIM data to estimate herd sizes at the end of 2013 (the year before the start of the outbreak). This was done by counting the number of animals in each herd in the national herd profile, a list of animals in every herd on the last day of the year, provided by DAFM. We did this to see if herds restricted during 2014–16 were typically larger or smaller than other herds with land in the study area.

Following Good and others [11] and Tratalos and others [12], we classifed Irish cattle herds as being of beef or dairy enterprise type in a given year if ≥66% of their stock were from beef or dairy breeds, respectively, calculated using their 2013 end-of-year herd composition. All other herds were classified as ‘mixed’. We used this information to examine whether herds restricted during 2014–16, and study area herds in general, were typically of dairy, beef or mixed enterprise type.

AIM movement data were also linked with information from AHCS on the bTB testing status of all Irish herds during 2013–2016, to examine the degree to which bTB restrictions could be explained by movements from infected herds outside the study area. For each herd in the study area during 2014–2016, we calculated whether any cattle which were introduced to the herd had themselves come from a herd which had tested positive for bTB. In this analysis, we obtained separate results with reference to two criteria for each of two measures: 1. the number and type of reactors in the source herd (the herd that the introduced animal was coming from), and 2. the time period during which the source herd might have tested positive. These alternative criteria were as follows:

- *For the number and type of reactors in the source herd:* 1a. the herd had at least one reactor (standard or inconclusive), or had a carcass identified with bTB-related lesions at slaughter, and 1b. a more restrictive definition, requiring ≥2 standard reactors.
- *For the time period during which the source herd might have tested positive:* 2a. *(a broader definition)* the selling herd had tested positive for bTB in the previous, same or next year, and 2b. *(a more restrictive definition)* as for 2a. but with positive test status in the next year not included. For the 2016 data, 2a. could not be employed, as at the time of this study we did not have movement or bTB test status data for 2017.

## Results

### General comment

Most analyses were conducted using the definition of study area based on the herd number DED *(the first definition of study area).* This is logical given that this approach was used by field staff throughout the outbreak when applying additional testing and control measures. However, for those analyses where geographic information systems (GIS) software was required (specifically herd location, kernel density analysis, herd characteristics and animal movement), the definition of study area was based on land parcel location *(the second definition of study area).*

### Cattle

#### The study area

Using the first definition of study area (based on herd number DED), there were 256 study herds during the study period which had received one or more herd tests for bTB. Using the second definition of study area (based on land parcel location), there were 467 registered herds on the LPIS database with an average of 73% of their land being within the study area (range: 0.004 to 100%), including 319 herds with cattle during 2013–16. Of these, 300 herds (herd size: mean 37.7 cattle, median 23) had cattle at the end of 2013, including 280 beef herds (33.0, 20), 16 dairy herds (110.6, 106.5) and four mixed herds (79.2, 71.5).

#### Herd-level results

**Fig. 1:**
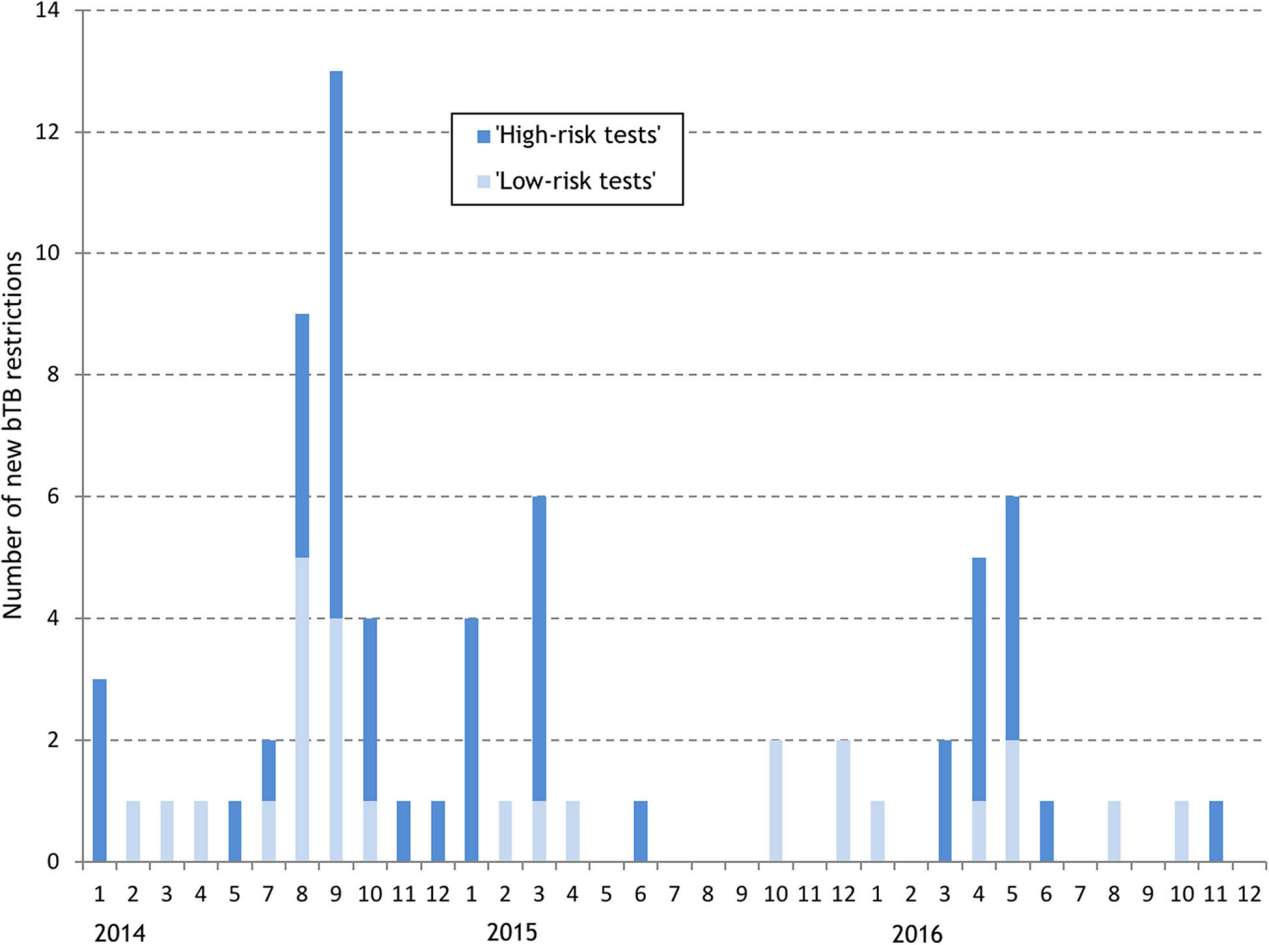
Number of new bTB restrictions in the study area each month during 2014–16, by risk classification of the breakdown test. Herds within the study area were determined based on the District Electoral Division of the herd number

**Fig. 2:**
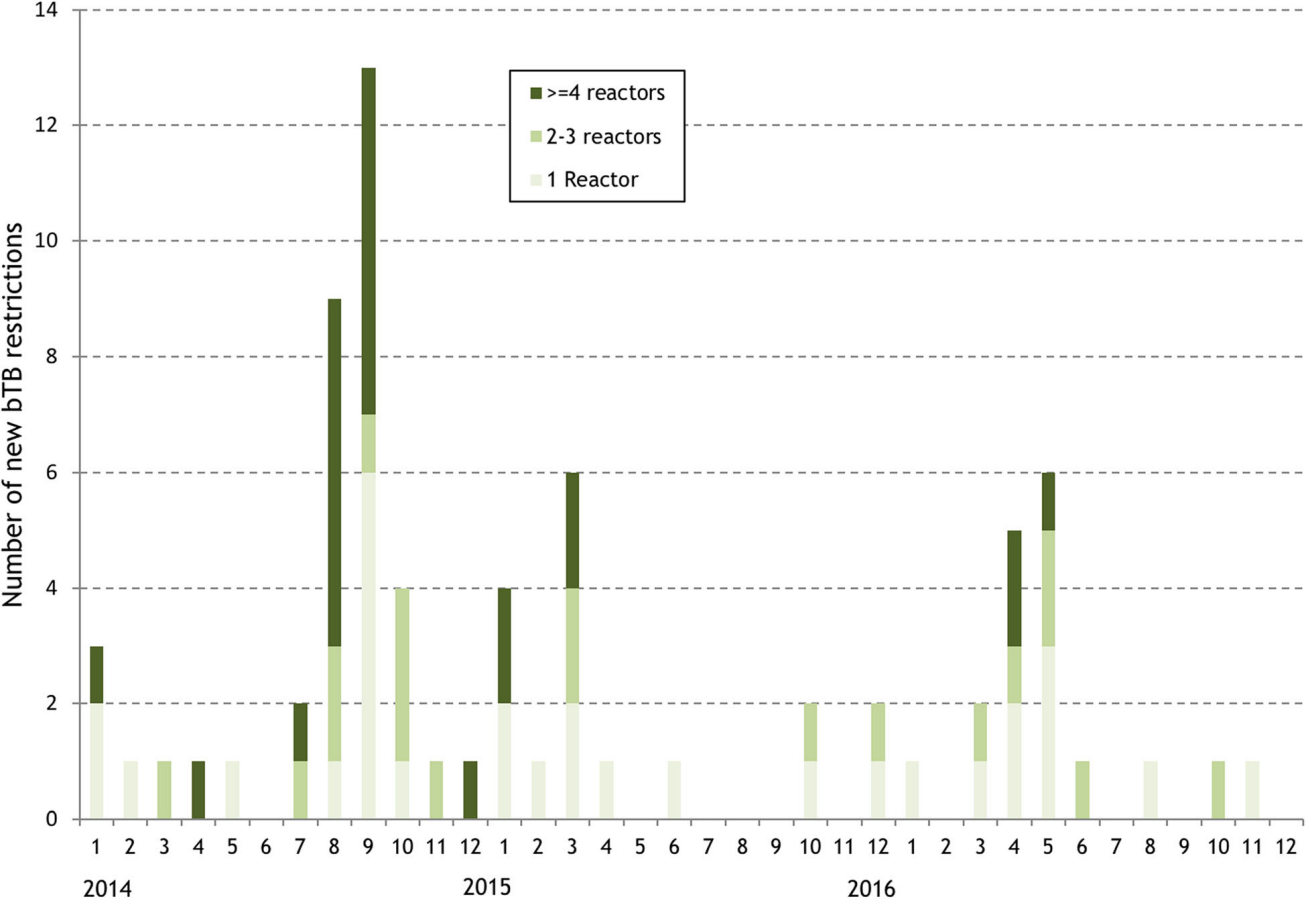
Number of new bTB restrictions in the study area each month during 2014–16, by restriction severity. Herds within the study area were determined based on the District Electoral Division of the herd number

**Fig. 3:**
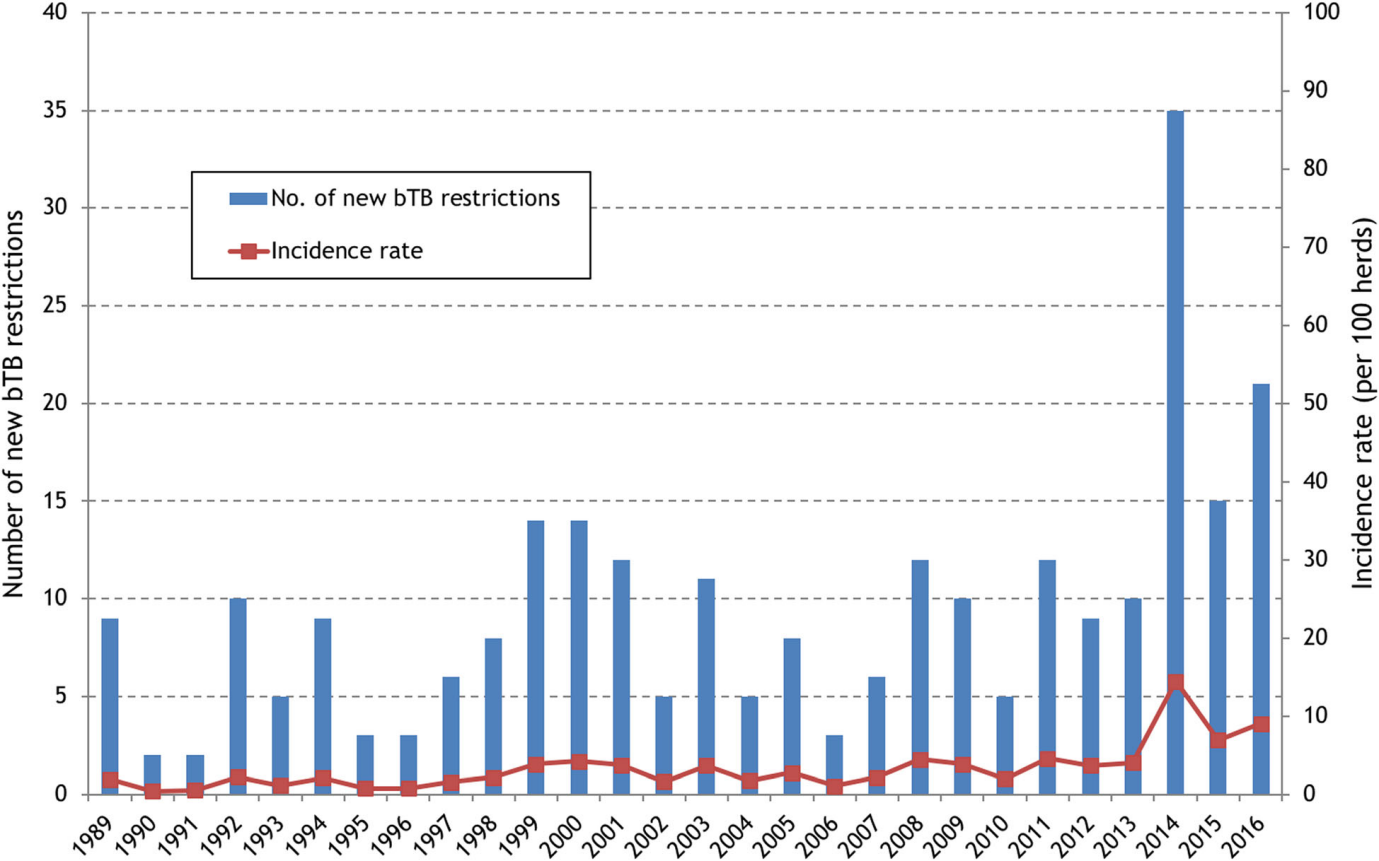
Number of new bTB restrictions and incidence rate (per 100 herds) in the study area during 1989–2016. Herds within the study area were determined based on the District Electoral Division of the herd number

Between 2014 and 2016, there were a total of 72 bTB restrictions in 65 study herds in the study area based on the herd number DED (Table 1). The majority of these (41.7%) had only 1 reactor/animal with a lesion, however, 23 (31.9%) had 4 or more reactors/animal with a lesion. Most (62.5%) of the restrictions began with a *high-risk* test (Table 1), and a higher proportion of restrictions starting with a *high-risk* test tended to disclose more (≥4) reactors (42.2% of *high-risk* tests versus 14.8% of *low-risk* tests). The majority of bTB restrictions began in 2014, with August and September having the highest number of new restrictions (Fig. 1). Fewer restrictions began in 2016, with most of these being less severe (Fig. 2). Prior to 2014, there had been less than 15 new restrictions per year (Fig. 3). In 2014, the number of new restrictions increased to 35, which was 14.5% of the study herds in the area affected (Fig. 3). Based on the land parcel location, there were an additional 9 bTB restrictions in the study area (a total of 81 restrictions in 74 herds).

**Table 1:** Number (%) of bTB restrictions, by restriction severity and risk classification of the breakdown test. Herds within the study area were determined based on the District Electoral Division of the herd number

Risk classification of the breakdown test	Restriction severity (Number of SICTT^a^ reactors/animals with bTB lesions at slaughter)			Total
	1	2–3	≥4	
*Low-risk* ^*b*^	14 (51.9)^c^	9 (33.3)^c^	4 (14.8)^c^	27 (37.5)^d^
*High-risk* ^*e*^	16 (35.6)^f^	10 (22.2)^f^	19 (42.2)^f^	45 (62.5)^d^
Total	30 (41.7)^d^	19 (26.4)^d^	23 (31.9)^d^	72

^a^Single intradermal comparative tuberculin test

^b^The initial breakdown test was classified as *‘low-risk’* if conducted on unrestricted ‘low’ risk herds. All unrestricted ‘low’ risk herds are tested annually with single intradermal comparative tuberculin tests (SICTT) conducted on all animals > 6 weeks of age (the annual test; test type [TT] 1)

^c^% of restrictions that were first detected with a *low-risk* test

^d^% of all restrictions

^e^All other breakdown tests using SICTTs were defined as *‘high risk’* tests, including inconclusive retests (TT3), contiguous (TT8), high risk (TT5A/F), post de-restriction (TT7B) and factory lesion tests (TT9A and TT10A; that is, following detection of a factory lesion during abattoir surveillance)

^f^% of restrictions that were first detected with a *high-risk* test

**Fig. 4:**
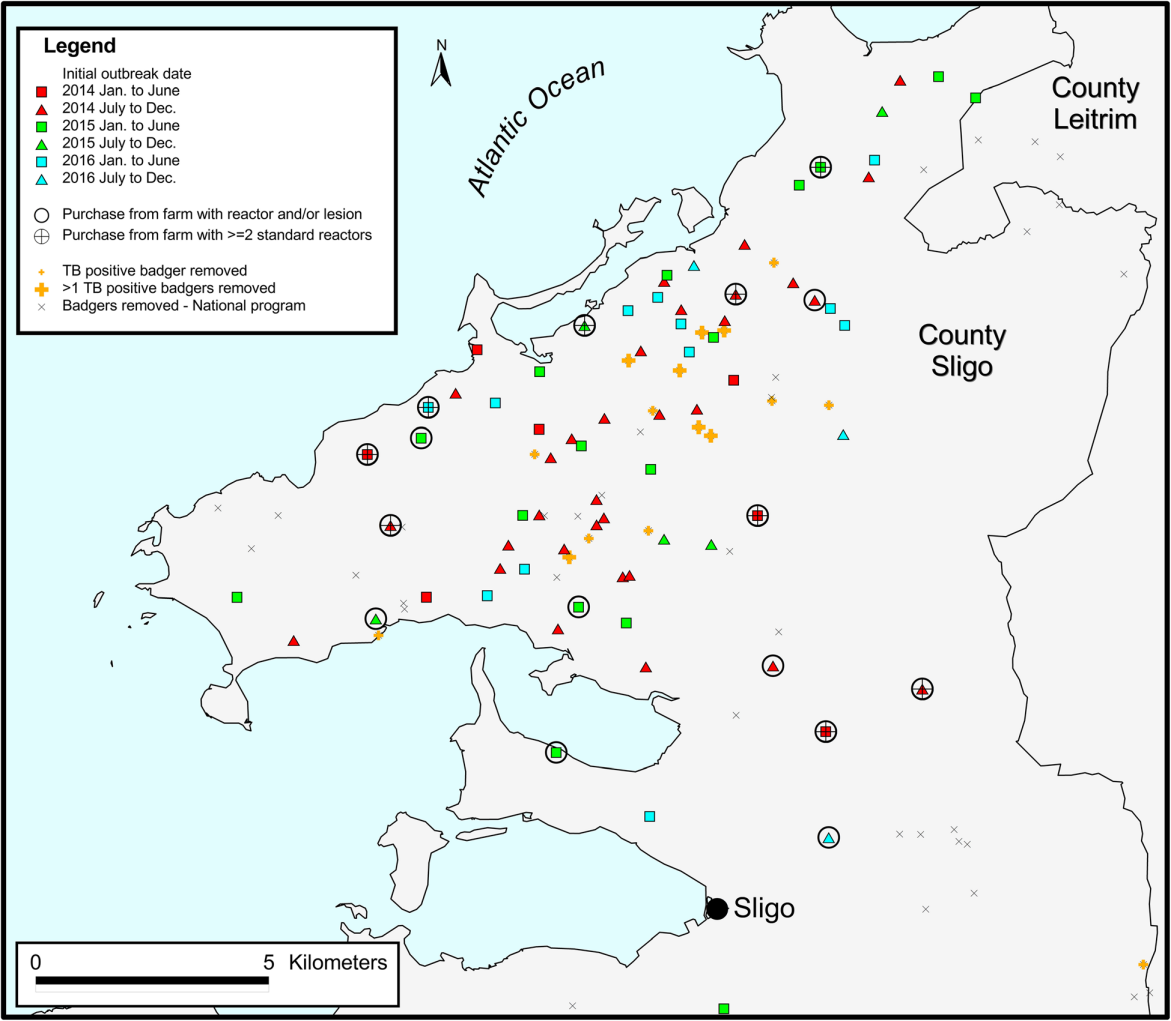
The location of restricted herds during 2014–16. The colour coding and dates refer to the time of the initial bTB restriction of each herd. Repeat herd restrictions during this period are not included. The black hollow circle represents herds where 1 or more ‘total reactors’ or a factory lesion had been discovered in a sending herd in the previous, same or next year, relative to the year of the discovery of infection in the receiving herd. The black hollow circle with a cross in the middle represents herds where 2 or more standard reactors were discovered in a sending herd in the previous, same or next year, relative to the year of the discovery of infection in the receiving herd. In each case, for herds that were first restricted in 2016, the 2017 data for ‘next year’ were not included. The small and large orange cross indicates that one or 2 or more bTB-positive badgers (positive on culture), respectively, had been removed from that location during 2014–16. The ‘x’ represents badger removals conducted through the national programme

**Fig. 5:**
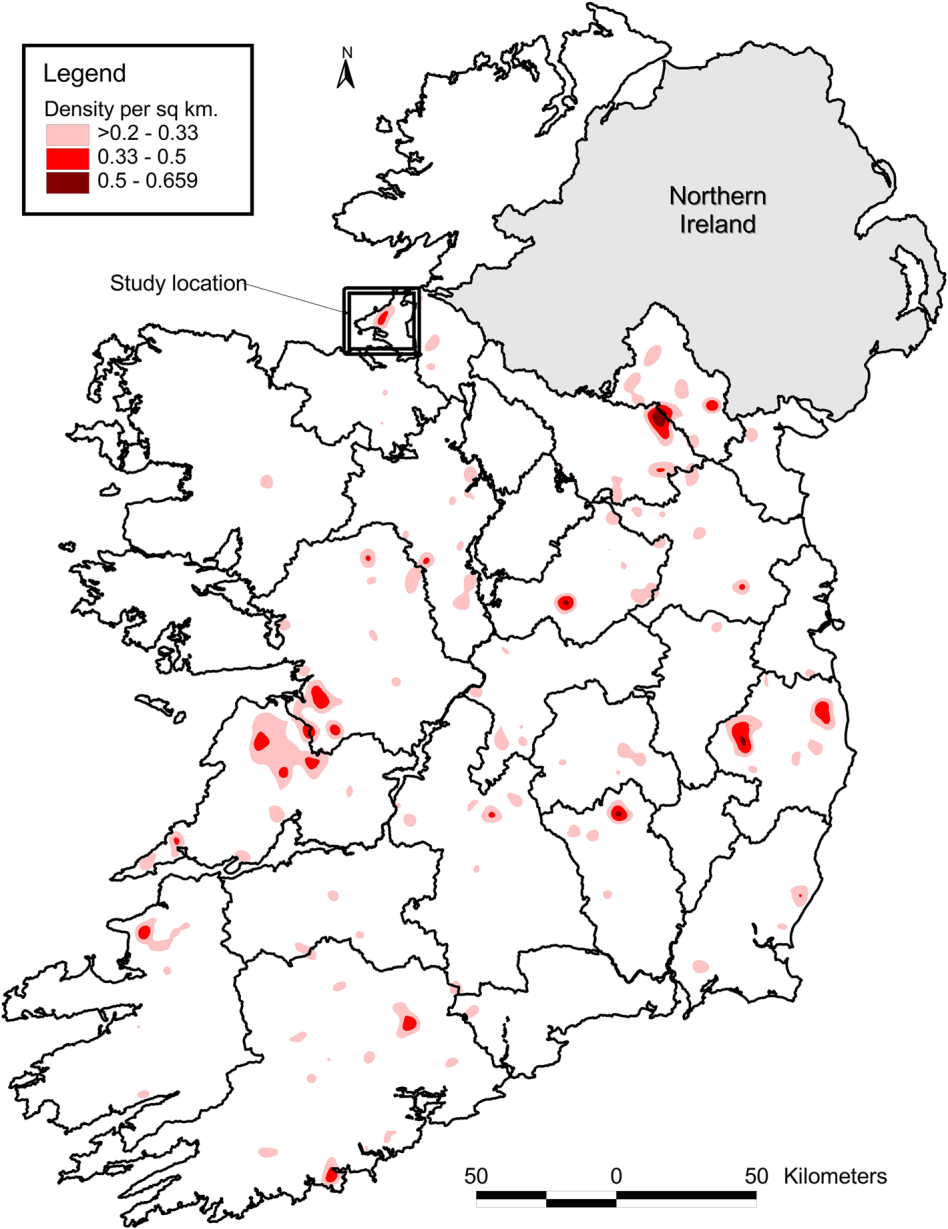
Kernel density analysis of all herds in Ireland with 2 or more standard reactors to the single intradermal comparative tuberculin test (SICTT) during 2014–16. The values represent the number of ‘positive’ herds per square kilometre. The location of north Co. Sligo is highlighted

The location of these study herds in the north Sligo area is presented in Fig. 4, and the kernel density analysis for all Irish herds during 2014–16 in Fig. 5.

A higher proportion of herds that were restricted in 2014–16 had a previous restriction in each of the 3 time periods shown in Table 2 compared to other herds in the study area. However, the proportion of herds with a previous restriction was only significantly (*p* < 0.001) higher for previous restrictions occurring between 2004 and 2008.

**Table 2:** Previous history of bTB restrictions in herds restricted during 2014–16 compared to other herds in the North Sligo study area. Herds within the study area were determined based on the District Electoral Division of the herd number

Herd bTB status during 2014–16	Herds tested between 2009–13	% of herds with a previous restriction in 2009–13	Herds tested between 2004–08	% of herds with a previous restriction in 2004–08	Herds tested between 1989–2003	% of herds with a previous restriction in 1989–2003
Not restricted	184	15.8	180	6.7	179	26.8
Restricted	65	20.0	65	24.6	62	33.9
*P*-value		0.433		< 0.001		0.289

*P*-value is based on a chi-square test of the difference between restricted and non-restricted herds in the percentage of herds with a previous restriction in the relevant time period

#### Animal-level results

The majority (55.5%) of the 387 test-positive animals were identified as standard reactors to the SICTT, with a further 29.5% positive to a diagnostic IFN-γ test (Table 3). Only 9 (2.3%) animals were first identified during abattoir surveillance. A further 14 animals (in addition to the 387 in Table 3) were removed as reactors either with clinical signs or as a result of their heightened risk of bTB exposure. These animals were SICTT negative (that is, the skin test difference was 0 or below the criteria for a severe inconclusive reaction), IFN-γ negative, and had no lesions detected at post-mortem. During the outbreak, two animals presented with clinical signs suggestive of bTB. A 3 month old calf presented with snoring but was otherwise in good health. At post-mortem, bronchial and mediastinal lymph nodes were enlarged and bTB was subsequently confirmed. Based on information provided by the herd keeper, an adult bull had failed to thrive over a period of approximately 12 m, presenting with cough and inappetence, however, bTB was not subsequently confirmed.

**Table 3:** Number (%) of test-positive animals in the study area during 2014–16, by type of disclosing test and year of test. Herds within the study area were determined based on the District Electoral Division of the herd number

Type of disclosing test	Year of test			Total
	2014	2015	2016	
SICTT^a^				
Severe inconclusive	4	1	2	7 (1.8)
Standard inconclusive	22	7	5	34 (8.8)
Standard reactor	146	27	42	215 (55.5)
Interferon (IFN)-γ test				
Diagnostic	107	7	0	114 (29.5)
Quality control^b^	7	1	0	8 (2.1)
Lesion found at slaughter	4	3	2	9 (2.3)
Total	290	46	51	387

^a^Single intradermal comparative tuberculin test

^b^For these 8 animals, IFN-γ was conducted in the context of diagnosis (all animals were negative to the SICTT), but was incorrectly conducted within 24 h (rather than within 8 h) of sample collection

**Fig. 6:**
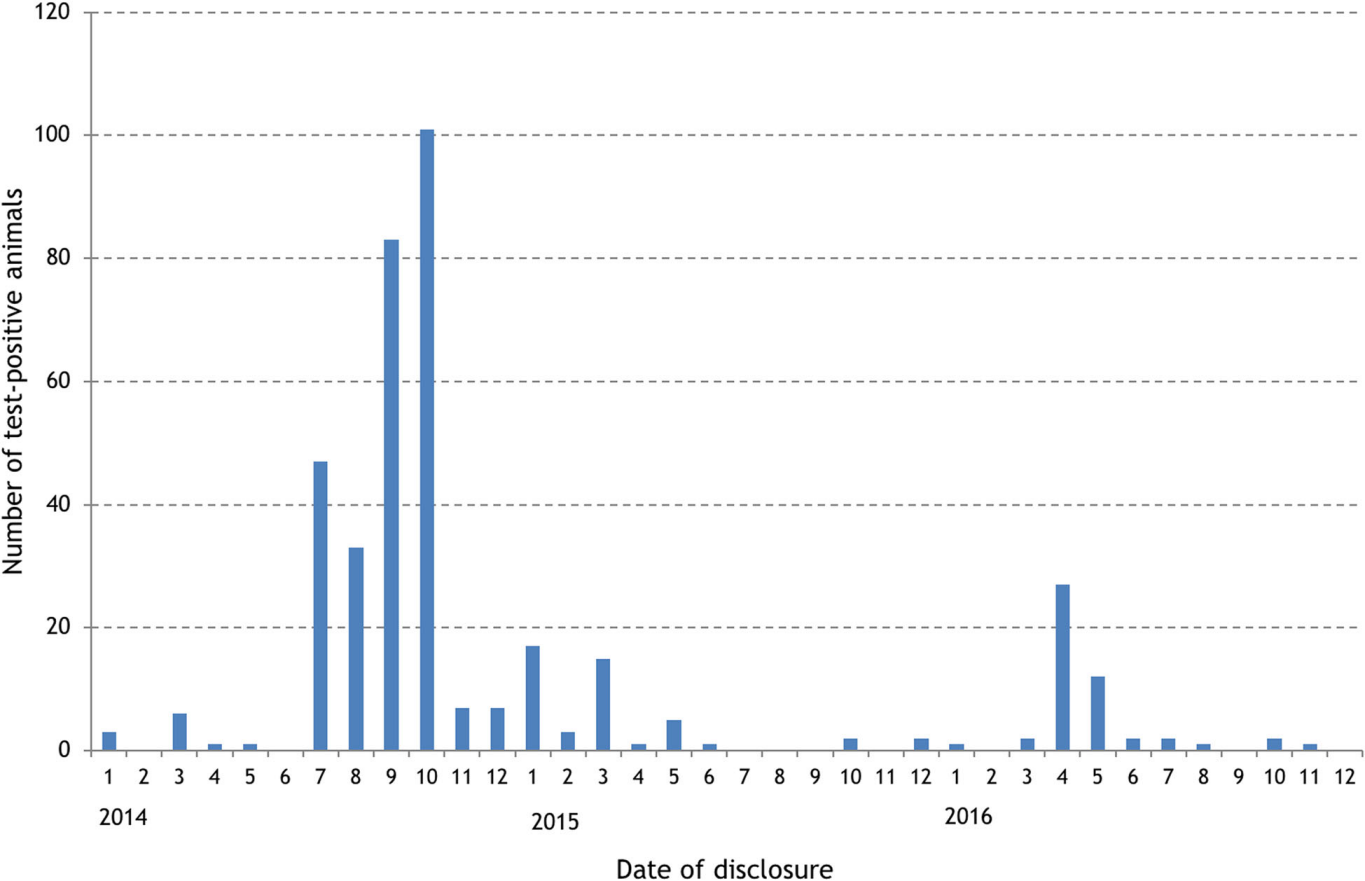
Number of test-positive animals identified during 2014–16 in the study area, by month of disclosure. Herds within the study area were determined based on the District Electoral Division of the herd number. In October 2014, one herd (Herd X) with only 2 SICTT reactors had interferon (IFN)-γ applied to the SICTT-negative animals with 59 testing IFN-γ positive

The number of test-positive animals peaked in September and October 2014 (Fig. 6). In October 2014, one herd (known as Herd X) with only 2 SICTT reactors had IFN-γ testing conducted on the SICTT-negative animals, of which 59 tested IFN-γ positive. However, none had a positive SICTT subsequent to the IFN-γ test. Between 2014 and 2016, 466 diagnostic (8-h) IFN-γ tests were conducted on animals within 60 days of an SICTT (Table 4). Of these, 24.5% were positive to the IFN-γ test. Of those positive to the IFN-γ test and subsequently slaughtered, 9.8% were positive at post-mortem, compared to 1.9% of those negative to the IFN-γ test. When Herd X was excluded, 16.5% were positive to the IFN-γ test. Of those positive to the IFN-γ and subsequently slaughtered, 16.4% were positive at post-mortem compared to 2.4% of the IFN-γ negatives (Table 4). Of the standard reactors that were tested using a quality control (24-h) IFN-γ test, 95.1% were positive. Of those IFN-γ positives and subsequently slaughtered, 66.3% were positive at post-mortem compared to 22.2% of the IFN-γ negatives.

**Table 4:** Information about animals tested using the interferon (IFN)-γ test, including number of tests, number of animals slaughtered and number (%) positive at post-mortem, by test type (diagnostic or quality control) and test result in the study area during 2014–16

Type of IFN-γ test, time difference between SICTT and IFN-γ	IFN-γ result	No. of tests (% positive)	No. slaughtered	No. (%) positive at post-mortem
Diagnostic IFN-γ test				
All herds	Negative	352	106	2 (1.9)
	Positive	114 (24.5)	92	9 (9.8)
All herds except Herd X^a^	Negative	278	83	2 (2.4)
	Positive	55 (16.5)	55	9 (16.4)
Quality control IFN-γ test	Negative	9	9	2 (22.2)
	Positive	174 (95.1)	169	112 (66.3)

Diagnostic IFN-γ tests were conducted on animals with a negative single intradermal comparative tuberculin test (SICTT) result based on an SICTT conducted no greater than 60 days prior to the IFN-γ test, whereas quality control IFN-ɣ tests were conducted on SICTT standard reactors. Herds within the study area were determined based on the District Electoral Division of the herd number

^a^In October 2014, herd X, with only 2 SICTT reactors, had IFN-γ testing applied, of which 59 animals tested IFN-γ positive. None of these animals had a positive SICTT subsequent to the IFN-γ test, and it is likely that these animals were all false-positive

**Fig. 7:**
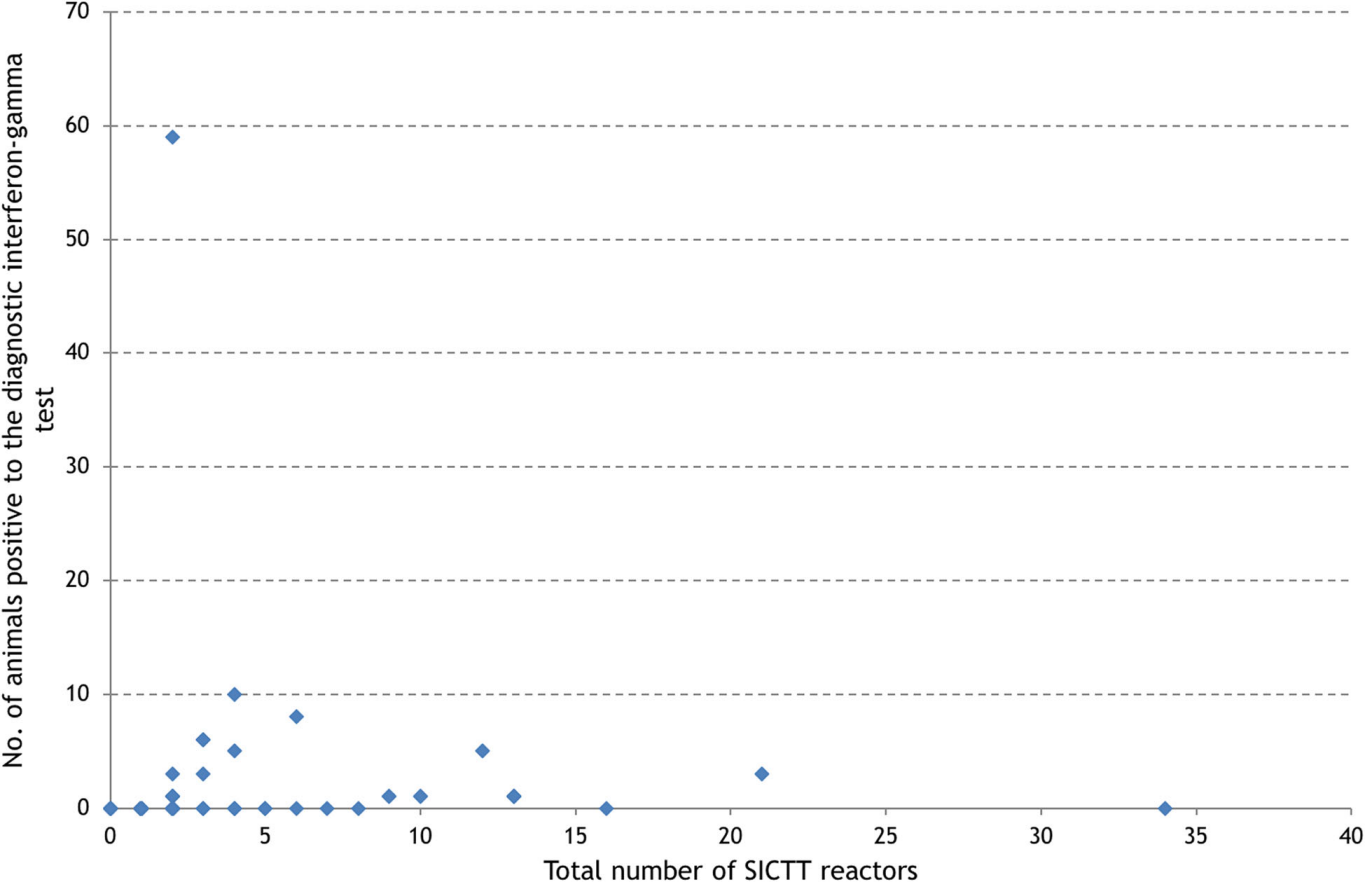
Number of test-positive animals that were identified in the study area using the diagnostic interferon-γ test during 2014–16, by the total number of SICTT reactors (standard, standard inconclusive and severe inconclusive) in the herd. Herds within the study area were determined based on the District Electoral Division of the herd number

The number of animals positive to the diagnostic IFN-γ test (and negative to the SICTT) per herd was 10 or less for all herds except Herd X (Fig. 7). The number of IFN-γ positives per herd did not appear to be related to the total number of SICTT reactors per herd.

#### Herd characteristics and movements

**Fig. 8:**
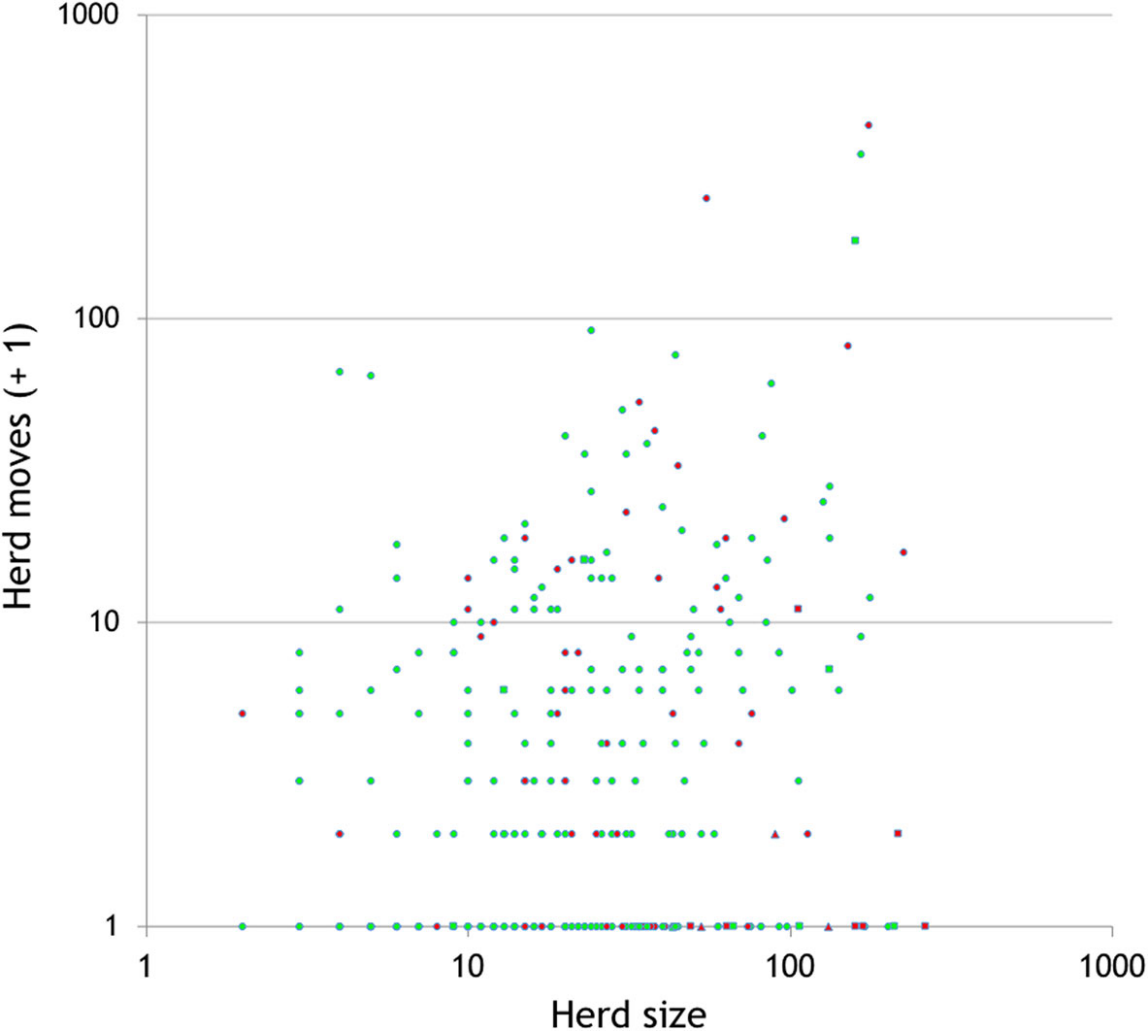
Relationship between herd size at the end of 2013, herd moves during 2013 and herd bTB status (restricted during 2014–16, not) for animals in the study area. Herds within the study area were determined based on the location of land parcels. Herd size and number of moves are both shown on a log scale, with a value of 1 also added to the moves data, to allow the display of zero values. Herd bTB status (): ○ Beef □ Dairy Δ Mixed

Herds restricted during 2014–16 were predominantly of beef enterprise type, similar to other herds in the North Sligo study area. However, a larger percentage of the restricted study herds were of dairy type or mixed (7 dairy, 3 mixed out of those of the 72 study herds restricted during 2014–16 which also had animals at the end of 2013) compared to other herds (9 dairy, 1 mixed out of 228 herds that were not restricted and had animals at the end of 2013). The 72 study herds restricted during 2014–16 were typically larger than other herds in the study area (mean: 52.4 vs. 33.1 bovines at the end of 2013) and introduced more animals during 2013 (16 vs. 8.3, respectively). Figure 8 presents a scatter plot of the number of inward animal movements during 2013 compared to study herd size at the end of 2013, by herd type (beef/ dairy/mixed) and bTB status (restricted during 2014–16/not restricted).

**Fig. 9:**
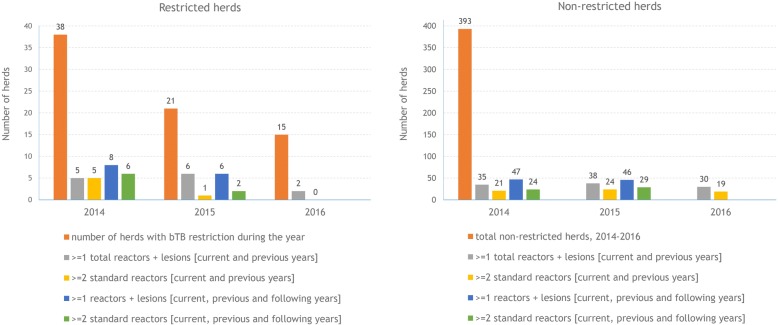
Number of herds in the study area with a bTB breakdown during each year during 2014–16 (left, orange bar), and number of other herds in the study area (right, orange bar), alongside 4 indicators of risk of bTB infection from the movement of cattle, calculated for each herd with reference to whether it had received animals from other herds previously or in the future containing reactor animals or with lesions discovered at slaughter. Only the first bTB restriction was included for seven of the study herds which had two restrictions during the study period. Indicators using data for the year following the year measured are not included for 2016, as data were not available for 2017. Note that the right-hand figure is scaled so that the bar for the total number of non-infected herds, 2014–2016, matches the total number of herds restricted during 2014. Herds within the study area were determined based on the location of land parcels

Herds restricted in north Co. Sligo engaged in only slightly more ‘risky’ inward movements than herds in the same area that were not restricted in terms of the average number of animals moving from bTB infected herds in the year previous, current or following the year of bTB restriction (Fig. 9).

### Wildlife

#### Badgers

In total, 145 badgers were submitted to Sligo RVL between autumn 2014 and the end of 2016 (Table 5). The locations of the badger removals and their bTB status are presented in Fig. 4. The locations of badger road casualties were not mapped.

**Table 5:** The number of badgers submitted to the Sligo Regional Veterinary Laboratory from the study area between Autumn 2014 and the end of 2016, including the number (%) that were road casualties, and the number (%) with visible lesions or positive on culture. Herds within the study area were determined based on the District Electoral Division of the herd number

	2014	2015	2016	Total
Total badgers	46	63	36	145
Road casualties	8 (17.4)	19 (30.2)	5 (14.0)	32 (2.1)
Visible lesions	6 (13.0)	3 (4.8)	4 (11.1)	13 (9.0)
*M. bovis* positive on bacteriological culture	9 (20.0)	17 (27.0)	12 (33.3)	38 (26.2)

#### Deer

In total, 17 deer were tested, with all found to be bTB negative.

### Response

During the outbreak, the primary aims of Sligo DVO staff were to identify infected herds as quickly as possible, to ensure that all infected animals in those herds were identified and removed, and to reduce the risk of infection from wildlife reservoirs of infection. In addition, any contiguous herds that had not been tested in the 4 months prior to being identified as contiguous herds were restricted to prevent animal movement from the infected area.

Initially, the high-risk area was defined as Sligo DEDs U101 to U109, excluding U103 and U108. Subsequently, the following steps were put in place:

- A management team was established to monitor progress. This team consisted of two area Veterinary Inspectors (VIs), supported by a further two VIs who were allocated to help with the extra workload.
- the Superintending Veterinary Inspector (SVI), the District Superintendent of Technical Agricultural Officers (TAOs), members of the wildlife unit, and Supervisory Administrative staff. While there was constant informal interaction, there was a formal meeting once every 2 weeks in the RVO to review progress.
- All reactor herds in the study area were considered high-risk herds and subjected to an epidemiological investigation.
- Testing of contiguous herds was given high priority. Normally, a contiguous programme is implemented for herds with three or more reactors, with the VI identifying the infected fragment and any herd with bovine animals within a 25 m buffer of this fragment being listed for testing. In the study area during the outbreak, all restricted herds were considered to be high-risk and a contiguous programme was set up for each restricted herd. The buffer for contiguous herds was increased to 50 m and all contiguous herds, even those outside the study area, were put on a contiguous programme. Any herd listed for a contiguous test that had not been tested in the previous 4 months was restricted immediately, to prevent infected animals moving out of the study area.
- There was increased use of diagnostic IFN-γ testing in restricted herds with two or more reactors in the study areas. All animals with high bovine readings, as well as epidemiological groups where reactor animals were located, were tested. Whole herd testing of all animals over 6 months of age was carried out if warranted.
- Reactor animals were IFN-γ tested for quality control purposes, when logistically possible, in order to assess the correlation between skin testing and IFN-γ results.
- Wildlife work, starting with activity surveys, commenced in September 2014. There was consultation with DAFM’s wildlife unit and, given the serious nature of the outbreak, it was agreed that a badger licence would be issued for any herd in the study area. All badgers caught were to be delivered to the local RVL for a full post mortem and culture. It was also agreed that there would be two rounds of badger capturing in the study area, in autumn 2014 and spring 2015. The badger capture programme was designed to eliminate the possibility of perturbation [13].
- In order to identify any role of deer in the breakdown, NPWS were asked to provide information on deer activity. A programme was put in place with local hunters, who would shoot deer and retain the head and red offal, to be brought to the RVL for gross examination and culture.
- Individual meetings were held with all private veterinary practitioners (PVPs) who conducted bTB tests in the study area, to update them on the situation and encourage high quality testing.
- Any possible clinical cases of bTB were to be euthanized and brought to the RVL for post mortem examination.
- Tissue from reactor animals and badgers testing positive for bTB was collected and sent to the Central Veterinary Research Laboratory (CVRL) to allow for strain typing in the future.

The north Sligo study area contains approximately 89 known badger setts, and badgers have been captured at 55 of these. Badger captures were made in the study area in November 2014, March 2015, March 2016, November 2016, and March 2017. This involved the creation of 16 work blocks, capturing 130 badgers, which included some road kills. The estimated number of person-hours involved in this operation was 1696 (staff of the Farm Relief Service: 16 blocks at 56 h per block (896 h); DAFM staff: 2 staff at 50 h per block (800 h)).

## Discussion

This paper describes a substantial outbreak over a 3-year period. A coordinated area-based approach was a key feature of the outbreak, and substantial resources were applied to bring the outbreak under control.

As yet, no definitive source was identified, nor reasons why a substantial number of herds were infected over a relatively short period. Past bTB history may have played a role, as bTB has been endemic in Ireland for many decades, including in north Co. Sligo during the years prior to 2014. As illustrated in Table 2 and in comparison to herds that were not restricted during 2014–16, a consistently higher percentage of herds restricted during 2014–16 had previous restrictions in each of the time periods examined: 20.0% (compared with 15.8% of other herds in the study area) in 2009–13, 24.6% (6.7%) in 2004–08, and 33.9% (26.8%) 1989–2003, although these differences were only statistically significant in 2004–08 (*p* < 0.001). Herd-level bTB risk can persist for many years following the derestriction of high-risk herds [14], attributable either to residual infection in cattle or reinfection, either from local sources (such as spread from environment, wildlife or farm-to-farm) or following cattle introduction [7]. Work presented in Figs. 8 and 9 provide insights into cattle movement, and its potential contribution to the outbreak. Herds restricted during 2014–16 received proportionally more bovine movements than other herds in the North Sligo study area, however, this might be expected given their typically larger size (Fig. 8). Herds testing positive in 2014 were slightly more likely to have received animals from herds which had or were about to test bTB positive, however, this was not the case for herds testing positive in 2015 and 2016 (Fig. 9). These comparisons can only provide clues as to the role of cattle movement, given that herds are tested at least annually, and therefore many herds will continue to trade for long periods after containing a bTB positive animal. Similarly, an infected animal may move between herds if anergic to the SICTT or not present in these herds at the time of the routine annual herd test. Previous work on this issue is conflicting, with Clegg et al. [15, 16] suggesting a limited role of animal movement in new herd restrictions, whereas several authors [7, 17] highlight the risk of infected but undetected animals in herds at the time of derestriction. The risk of residually infected animals at the time of herd derestriction is influenced by current EU legislation [18], which allows herds to return to trade within 2 clear full-herd tests, equivalent to 4 months of removal of the last known infected animal. In the absence of whole genome sequencing (WGS) or other methods of genetic discrimination, a technique now widely used in other jurisdictions [19, 20] but not yet in routine use in Ireland, we were unable to link this outbreak to those in other regions, including previous bTB clusters in neighbouring countries, both in Ireland and Northern Ireland. Badgers are recognized as a reservoir and important contributor to the epidemiology of bTB in cattle in Ireland [2, 21], and there was close spatial association between infected cattle herds and badgers (Fig. 4). Unfortunately, however, the badger *M. bovis* data provides no additional insights (e.g. information about directionality, as might be possible if WGS were available) relevant to the epidemiological role of badgers in the outbreak. Recent examples of the value of WGS to investigate bTB outbreaks such as this are available from several countries, including New Zealand [22] and the USA [23].

A coordinated regional approach was introduced shortly after the start of the outbreak, particularly with respect to contingency testing and the use of IFN-γ in known infected herds. Therefore, the trajectory of the outbreak (for example, the bTB incidence rate in Fig. 3, the number of new bTB restrictions in Figs. 1 and 2) has been greatly influenced by both the schedule and intensity of herd testing during 2014–16. As highlighted in Figs. 1 and 2, a substantial proportion of new bTB restrictions were identified through *high-risk* testing, such as contingency testing, which reflects the increased intensity of field surveillance, for example through contiguous testing. Further, many of the new bTB restrictions, particularly in 2014, but also in 2015 and 2016, included multiple test-positive animals. As expected, given this management emphasis, the majority (55.5%) of test-positive animals were identified as standard SICTT reactors, with the balance being detected through use of the IFN-γ test (31.6%), as standard SICTT inconclusive reactors (8.8%), during abattoir surveillance (2.3%), or as severe SICTT inconclusive reactors (1.8%) (Table 3). In Ireland, the IFN-γ test is used both to detect infected but SICTT-negative animals (the diagnostic IFN-γ test, testing is conducted within 8 h of sample collection) and as a means to quality control SICTT-positive animals (the quality control IFN-γ test; testing is conducted within 24 h of sample collection). In this bTB cluster, the IFN-γ test allowed the identification of 122 test-positive animals (Table 3), including a number that were positive at post-mortem. Apart from Herd X, in comparison to the number of SICTT reactors, there were relatively few IFN-γ test positive animals (Fig. 7). In this study, it is not possible to quantify the additional value of using the IFN-γ test, however, results of earlier work highlighted the risk associated with retaining IFN-γ positive animals that were negative to the SICTT. In this earlier work, Clegg et al. [14] concluded that prompt removal of these animals is necessary to reduce the potential for future transmission. However, it is important to note that there are dangers in the application of the IFN-γ test in low-risk situations, that is in herds not meeting the criteria of 4 reactors. Herd X, with only 2 reactors, had IFN-γ testing applied with many subsequent test-positives. Based on subsequent follow-up, it is likely that these animals were all false-positive.

The map shown in Fig. 5 illustrates a number of similar bTB clusters across Ireland, including some with a higher concentration of bTB reactors per km^2^ than in the North Sligo cluster. This does not diminish the significance of the management of the Sligo event, but rather suggests that lessons learned in Co. Sligo may be of relevance elsewhere. This kernel density map needs to be interpreted with some caution. Firstly, it doesn’t take farm density into account; in the absence of a denominator, it is essentially a density of qualifying herds. Secondly, the map is restricted to the time interval covered by the North Sligo cluster. In contrast, the other clusters could be a continuation of existing clusters, a large event in a single year which has ended, or an emerging event. Finally, the search radius of 5 km that was used in the analysis was selected to identify local-level clustering. To have a clearer understanding of what constitutes a true bTB cluster in Ireland, an in-depth study would be required.

In this work, two different definitions of the study area have been used. Throughout the outbreak, the DVO defined the study area using information gathered from the herd number. In Ireland, the herd number includes an indication of the DED to which it is associated. We also used this approach for most analyses, to ensure that study results were directly relevant to the key decisions that influenced regional disease control. Nonetheless, the DED assignment of herd numbers is imperfect, particularly when account is taken of the fragmented nature of Irish farms. During GIS-associated analyses, it proved more logical to define study area based on land parcel location. These differing approaches need to be considered during study interpretation.

There were a number of lessons learned during management of the North Sligo bTB cluster, as outlined below:

- It is important to create a sense of urgency around significant bTB outbreaks, to ensure that infected herds and infected animals within herds are detected as soon as practicable.
- It is critical that there is a team in place to manage the outbreak, with each team member understanding the overall objective in managing the outbreak, and their role in the team response.
- Ensure that all team members who interact with the public and with farmers is delivering a consistent message.
- Treat the problem as an area problem, noting that all breakdowns are of importance.
- A degree of flexibility is important for the breakdown manager, enabling them to deploy resources as required.
- Additional tools are needed, particularly molecular methods, to assist in answering key questions relating to the source and spread of *M. bovis* to many herds during this bTB outbreak.

## Conclusions

This paper describes a substantial bTB outbreak in north Co. Sligo during 2014–16, the response that was undertaken and some lessons learned. A coordinated area-based approach was a key feature of the outbreak, and substantial resources were applied to bring the outbreak under control. There remain important unanswered questions about the outbreak, relating to the source and spread of *M. bovis* to many herds in this region. There may be linkage with previous clusters, however, this is currently speculative. Additional tools are needed to answer these questions, including the routine application of molecular methods such as WGS. A coordinated regional approach was taken, and a number of lessons were learned.
